# Asian American University Students’ Experiences during the COVID-19 Pandemic: A Qualitative Study

**DOI:** 10.3390/bs14010034

**Published:** 2024-01-03

**Authors:** Jacqueline Hwang, Yi Ding, Cixin Wang, Eric Chen, Ying Wu, Xiaoyan Hu

**Affiliations:** 1 Graduate School of Education, Fordham University, New York, NY 10023, USA; jhwang26@fordham.edu (J.H.); echen@fordham.edu (E.C.); ywu135@fordham.edu (Y.W.); xhu118@fordham.edu (X.H.); 2 College of Education, University of Maryland, College Park, MD 20742, USA; cxwang@umd.edu

**Keywords:** COVID-19, pandemic, university students, Asian American, adjustment, coping, discrimination, identity

## Abstract

In addition to the unprecedented challenges and stressors that university students faced during the COVID-19 pandemic, Asian American students experienced specific hardships due to COVID-19-associated xenophobic attitudes, harassment, and assault against people of Asian complexions. This qualitative study aimed to explore the ways in which Asian American university students’ experiences during the pandemic changed their views of their identities as Asian Americans by analyzing in-depth interviews of four case study participants. Secondary analysis of two waves of interviews, which were conducted during the initial outbreak of the COVID-19 pandemic and during a six-month follow-up, and primary analysis of a newly conducted third wave one year after the initial outbreak yielded 12 themes that captured the essence of the Asian American university students’ experience and redefining of their identity during the pandemic. The four participants identified these themes across four categories: Experiences and Events during the Pandemic; Categorization of Asians in America; Confronting Asian Discrimination; and Renewed Sense of Identity. The longitudinal findings revealed direct experiences and perspectives regarding the influence of the COVID-19 pandemic on Asian communities, as well as the impact of the various social and political events during this time period, such as the Black Lives Matter Movement (2020) and the 2020 US presidential election. The implications, limitations, and future directions are discussed.

## 1. Introduction

As suggested by government records, the first person infected with COVID-19 might have been a 55-year-old Hubei resident in China. Wuhan, Hubei Province, China, quickly became the epicenter of the COVID-19 outbreak in November 2019, and COVID-19 spread rapidly and internationally [1,2]. Racism has been displayed through anti-Chinese and anti-Asian scapegoating for the spread of COVID-19, contributing to discriminatory behaviors against Asian people. These behaviors have been found at the individual level in daily interactions as well as in the media and public health discussions. Reported racialized incidents and acts of prejudice included bullying, harassment, violence, and discrimination. Hate crimes against Asian Americans rose exponentially across the United States in 2020 and continued throughout the pandemic [3,4]. In addition to the pandemic-induced stress and anxiety, those who were not subjected to hate and acts of violence still experienced mental fatigue and exhaustion as a result of the rising anti-Asian sentiment and xenophobia. Although there have been numerous studies examining Asians’ perspectives and experiences during the COVID-19 pandemic, very few studies have provided data from a longitudinal perspective. The societal impact of the COVID-19 pandemic evolved throughout its extended duration; thus, it is important to explore Asians’ experiences over a relatively longer period of time. A multiple case study analysis with a phenomenological approach was used in this study to explore Asian American university students’ unique lived experiences during the COVID-19 pandemic. The U.S. Census Bureau defines “Asian” as “a person having origins in any of the original peoples of the Far East, Southeast Asia, or the Indian subcontinent including, for example, Cambodia, China, India, Japan, Korea, Malaysia, Pakistan, the Philippine Islands, Thailand, and Vietnam.” Due to the current study’s researchers’ personal identities and networks, four Asian American university students with Chinese or Taiwanese backgrounds were recruited and included in this study.

### 1.1. Xenophobia towards Asian Americans

COVID-19 fueled xenophobic attitudes and actions against people of Asian complexions. Racism has been displayed through anti-Chinese and anti-Asian scapegoating for the spread of COVID-19, as well as reactions of fear, exclusion, microaggression, and other discriminatory behaviors towards both Asian children and adults [5,6,7,8,9,10,11,12,13,14]. Among other racist terms for the virus, President Trump repeatedly referred to COVID-19 as the “Kung Flu” [7,15]. Additionally, news reports of the coronavirus situation were accompanied by photos of Asians and Chinatowns. In multiple cases, the photos used in publications from outlets such as the *New York Times*, the *New York Post*, and *The Hill* were not directly related to coverage of COVID-19 and article contents [16]. The misuse of photos and other media reporting choices contributed to the public’s stigmatizing attitudes against Asians [16,17,18].

Because Asian Americans were being harassed and verbally and physically assaulted as a result of the racist attitudes surrounding the COVID-19 pandemic, various platforms have been launched for citizens to report hate crimes and bias-based incidents [12,13,19,20]. Received reports included bullying, harassment, violence, and discrimination against Asian Americans, and at least 2808 reports of anti-Asian discrimination were reported between 19 March and 31 December 2020 [12,13]. The Center for the Study of Hate and Extremism at California State University, San Bernardino (2021), reported that in 2020, hate crimes against Asian Americans rose by almost 150% in major US cities, despite overall hate crimes dropping by 7%. The New York Police Department (NYPD) reported that anti-Asian sentiment hate crimes increased by over 1900% in 2020 in New York City [11]. In January 2021, the newly inaugurated President Biden signed an executive order denouncing anti-Asian discrimination [21]. However, anti-Asian violence continued, and over 500 incidents were reported to the Stop Asian American and Pacific Islander (AAPI) Hate center in the first two months of 2021 [12]. It is important to recognize that these counts are not complete as many incidents go unreported, yet these incomplete data give the public some insight into the Asian and Asian American community experience during the COVID-19 pandemic. Many anti-Asian attacks go unreported for a number of reasons, according to Dr. Russell Jeung, who helped Stop AAPI Hate (i.e., language barriers, lack of trust that action will be taken if a report is made, cultural tendency to remain quiet) [22]. 

### 1.2. Prejudice

Merriam-Webster [23] defines prejudice as “an irrational attitude of hostility directed against an individual, a group, a race, or their supposed characteristics.” Prejudice is an adverse opinion or preconceived judgment formed without a fair basis or sufficient information [24,25,26]. “Racism” occurs when racial prejudice and racial dominance occur simultaneously. Racial prejudice occurs when there are inaccurate or negative beliefs that rationalize the superiority of one race. Racial dominance occurs when there is control of societal structures by a racial group, which maintains that group’s privileges [27,28]. 

According to Allport [24], there are five stages of prejudice. On Allport’s scale, ranked by increasingly produced harm, physical attack (i.e., hate crime) is ranked fourth. In the United States, hate crime is defined by the Federal Bureau of Investigation (FBI) as “criminal offense[s] against a person or property motivated in whole or in part by an offender’s bias against a race, religion, disability, sexual orientation, ethnicity, gender, or gender identity” [29]. Each stage of prejudice enables the next, and people only proceed to subsequent stages of prejudice if they received support for their behavior in earlier stages. Whether or not prejudice is displayed and carried out through actions depends on the surrounding social context and social norms [24,30,31]. Media and messages spread via social media have a role in prejudice and xenophobia. The increase in anti-Asian sentiment that spread throughout the country during the COVID-19 pandemic was fueled by scapegoating and encouragement of division: to view the Asian and Asian American community as members of an out-group due to fear. For example, a study analyzing tweets on the social media platform Twitter a week before and after President Trump’s first tweeting of the provocative rhetoric phrase “Chinese Virus” showed that anti-Asian sentiment rose during the week after his tweet: the percentage of tweets with anti-Asian sentiment using the hashtag #COVID-19 was 19.8%, compared to 50.4% with #Chinesevirus [32]. The stages of antilocution against, avoidance of, and discrimination against Asians and Asian Americans received support and encouragement from influential social figures (e.g., the President’s rhetoric), which, as Allport’s theory of prejudice explained, enabled this stage of physical attacks.

### 1.3. Myth of Asian Americans as a Model Minority

The model minority myth also contributed to the COVID-19 anti-Asian sentiment, as it distracts from the historical and continued discrimination that the group experiences. Asian Americans have been stereotypically labeled as the “model minority”, as law-abiding, model citizens who are polite, hard-working, family-oriented, and who can achieve a high level of success academically and economically. The term “model minority” appears to praise Asian Americans for their achievements, but the label can be harmful for the population because it carries high expectations for achievement and promotes the stereotype that they are quiet and submissive individuals who will not speak out or protest [33,34,35]. Furthermore, the oversimplification of the term “Asian American” groups together various distinct and different ethnic groups who differ in achieved income, education, and assimilation levels. Aggregate statistics overgeneralize and do not accurately represent all groups under one category [33,35,36]. 

The effect of the model minority myth is that Asian Americans are pit against other racial minority groups [33,34]. Additionally, because of this “model minority” status and prevalent Asian American stereotypes, the discrimination and racism experienced by Asian Americans can be more subtle, or misunderstood, than that directed towards other groups of people of color [37,38,39,40]. There is also a misconception that Asian Americans do not encounter major challenges because of their race, and the model minority myth has been used as supportive “evidence” that racial discrimination against this race does not exist [33,36]. The results of a qualitative study that examined the reactions of students and college administration at a university in response to racial incidents involving Asian American and African American students showed that reactions were comparatively less profound and immediate for racist incidents that involved Asian Americans [41]. The model minority label can cause indifference and even nonaction when Asian Americans are victims of racial intolerance and misrepresent the discrimination that the group faces [33,36,42].

The model minority myth obscures the socioeconomic and educational diversity within the Asian American community, and the stereotype maintains ignorance and distorted perceptions of the experiences of Asian Americans, especially college students [33,36]. Asian American individuals often experience pressure to conform to racial stereotypes, and if they do not live up to the model minority image of success, which many have internalized, there is a sense of failure [36,42]. Because of the myth and assumption that the model minority group can succeed with little support, Asian American students tend to not receive the same supportive resources and services as students of other minority racial groups, making them the “invisible minority” [35,42]. Because they are believed to be academically strong and not experience vulnerabilities, the model minority myth has caused the Asian American community to be understudied in higher education research [36,43,44,45].

### 1.4. Asian American Identity

Asian people’s view of themselves is influenced by what others think of them, an effect of the collectivistic culture, which means that their racial identity is affected by their social environment [46,47,48,49]. According to Bell’s oppression theory, Asian Americans are “historical subjects”, and their lives are shaped by historical and current events [50]. Asian American identity formation is the result of responding to the surrounding social contexts through both the internalization and externalization of environmental contexts, individual assumptions, relationships, and interactions. Asian Americans constantly reshape and redefine their own identities [48]. The Asian American identity is an integrated sense of the self, incorporating past and present cultural contexts [49]. According to racial identity development theory, identity conflict can be experienced during a period like the COVID-19 pandemic when Asian Americans are living in a context where there is institutionalized racism through societal structures, cultures, and values [46,47]. 

Kim’s Asian American Racial Identity Development Model [46] describes the process of Asian American identity in five conceptually distinct, sequential stages: Ethic Awareness stage, White Identification stage, Awakening to Social Political Consciousness stage, Redirection stage, and Incorporation stage. Throughout the lifetime, Asian Americans explore their ethnicity and identity through various common experiences, which are outlined as interactions with their family members and relatives; forming positive or neutral attitudes towards their ethnic origin; experiencing the tendency to assimilate to White society; adopting new perspectives associated with increased political awareness; understanding oppression and oppressed groups; reconnecting or renewing connections with Asian American heritage and culture; experiencing increased self- and group-pride, as well as anger towards white racism and oppression; and having a positive and comfortable identity as an Asian American, along with respect for other racial and cultural heritages [46,47]. 

### 1.5. Purpose of this Study

Asian American identity is influenced by social contexts, such as relationships and external forces [46,47,48,49]. This study served to gain a greater understanding of the experiences of Asian American university students during the COVID-19 pandemic via qualitative analyses using a multiple case study methodology with a phenomenological approach to explore the following question: How have Asian American university students’ experiences during the COVID-19 pandemic changed their view of their identity as Asian Americans? 

## 2. Method

### 2.1. Participants

The current study recruited participants from an original larger-scale study, which included both quantitative data collection and qualitative data collection. For the original study that quantitatively examined university students’ adjustment, coping, and stress during the COVID-19 pandemic, participants were eligible if they were at least 18 years old and enrolled in an American university at either the undergraduate or graduate/professional level during the initial peak of the COVID-19 pandemic. For the current study, interview data from Asian American students from wave one (during the initial peak of the pandemic) and wave two (six-month follow-up) of the original study and a newly collected wave three were used for analyses. In total, 12 Asian American students were interviewed during wave one; 10 of them responded to invitations and were interviewed during wave two and then were contacted for their interest in participating in wave three. Four of the seven interested participants were chosen based on the quality of their wave one and two interviews and their previously shared experiences regarding their Asian identification as related to the focus of the current study. Consideration was also given to their other areas of identification (e.g., gender, student status during wave three, field of study, location in the United States). The four Asian American participants chosen for wave three became case studies for the current study to explore the ways in which their pandemic experiences impacted their sense of Asian American identity. Interview data from waves one, two, and three of these multiple case study participants were used for qualitative analyses for the current study. 

### 2.2. Measures

The study utilized a combination of previously conducted interviews from a larger-scale study (i.e., wave one and wave two) and newly conducted interviews specifically for this study (i.e., wave three). All interviews were conducted in English and consisted of open-ended questions that aimed to facilitate the exploration and reflection of experiences during the pandemic. While wave one and two interviews were conducted as part of a larger-scale study to explore university students’ experiences and to highlight areas for support during the pandemic, the purpose of wave three for this study was to explore the ways in which Asian American university students’ pandemic experiences impacted their sense of identity as Asian Americans.

Included in this study were four interviews per time point, one from each case study participant during wave one, wave two, and wave three. Wave one interviews were conducted in April 2020. Audio-recorded Zoom interview lengths for wave one ranged from 16 to 26 min (M = 20.63, SD = 5.38). Wave two interviews were conducted in November 2020. Audio-recorded Zoom interview lengths for wave two ranged from 23 to 39 min (M = 30.63, SD = 6.45). Wave three interviews were conducted in June 2021. Audio-recorded Zoom interview lengths for wave three ranged from 51 to 76 min (M = 66.25, SD = 11.96).

### 2.3. Procedures

Institutional Review Board (IRB) approval was obtained from Fordham University for this study. The original larger-scale study also obtained IRB approval from Fordham University prior to data collection. All participation was voluntary, and electronic consent was obtained prior to study participation. All participant information was deidentified. Qualitative analyses were conducted on a collection of both secondary data collected during two time points of follow-up interviews for the original larger-scale study with the Asian American university students and newly collected wave three interviews that were specifically conducted for the current study. Interview respondents were recruited from the original larger-scale study’s participant pool, who voluntarily completed a Qualtrics survey. The survey participants were recruited through convenience sampling via professors’ and students’ professional networks across the United States. Those interested in participating in the follow-up interviews provided their contact information and availability at the end of the original survey. Using a random number generator, interview participants were selected based on their identified ethnicity, gender, field of study, and student status (i.e., undergraduate or graduate level, year of study, and domestic or international student). The interviews were conducted in a semi-structured, open-ended manner. Wave one and wave two interview participants of the original larger-scale study were compensated with a $10 Amazon gift card for their participation after each interview. Audio recordings from wave one and wave two of the interviews were saved and transcribed for the original larger-scale study. 

Interest was solicited from wave two participants for voluntary participation in wave three, and those who participated were compensated with a $20 Amazon gift card. Audio recordings from wave three interviews were saved and transcribed. Wave three interviews were also conducted in a semi-structured, open-ended manner, and elaboration of the participants’ responses was elicited. Verbal consent for participation in three waves of interviews was collected prior to starting audio recording. Interviews for all three time points were transcribed by the primary researcher and graduate assistants of the researcher’s mentor. Each interview transcript entered a multi-audit/review for accuracy by the research team. A deidentification process was applied, and pseudonyms were created to maintain the participants’ anonymity. Three waves of interview data were included in the current study. The questions used for each wave of interviews are included in Appendix A at the end of the article. 

## 3. Results

The qualitative aspect of the current study attempted to serve as exploratory, descriptive, and explanatory, examining the Asian American university student experience of COVID-19 from different perspectives to understand the phenomenon. A multiple case study methodology with a phenomenological approach was used to provide an understanding of the experiences of Asian American university students over the course of the COVID-19 pandemic. 

Specifically, Moustakas’s transcendental phenomenology method [51] was used to provide a description of the experiences of Asian American undergraduate and graduate students by highlighting significant statements (horizontalization). A systematic interpretation of interview transcripts and extraction of common themes across the interviews developed clusters of meaning from the four case study participants’ three waves of interviews about their experiences during the COVID-19 pandemic as Asian Americans [51,52]. Transcendental phenomenology is a philosophical approach to qualitative research methodology, and it seeks to understand human experience [51]. This approach often utilizes epoche or bracketing. Researchers examine phenomena by setting aside preconceived notions or biases and exploring the essence and structure of phenomena as they are experienced, which allows the intrinsic meanings to emerge within their own identities [51].

### 3.1. Descriptive Analyses of Variables

Participants ranged in age between 18 and 25 years (M = 23, SD = 3.37). Three participants identified as women, and one identified as a man. Three participants were located on the East Coast, and one was located on the West Coast. The participants’ specific locations were identified relative to the location of the university where the original study was conducted (Fordham University in New York City) as specific social, political, and personal experiences during the COVID-19 pandemic were impacted by geographical location. The New York City metropolitan area is defined as the city and suburbs of New York City, including Long Island and the Mid- and Lower Hudson Valley in New York, north and central New Jersey, three counties in western Connecticut, and five counties in northeastern Pennsylvania. Three participants indicated a household income of more than $100,000, and one between $20,000 and $49,000. All participants were second-generation, non-international Asian Americans. With regard to Asian ethnicity, three participants identified as Chinese, while one participant identified as Taiwanese. All participants had some language proficiency in Chinese. One participant was in her first year of undergraduate studies at the onset of the COVID-19 pandemic, while the other three participants were pursuing graduate degrees. Two of the participants were STEM students, one participant was studying in the medical field, and the other participant was studying in the social science field. See Table 1 for details on all four case study participants’ background and salient personal information. 

### 3.2. Qualitative Analysis

A multiple case study analysis with a phenomenological approach resulted in the identification of 12 common themes across four categories that captured the essence of the Asian American university students’ experiences during the COVID-19 pandemic that affected their view of their Asian American identity. Interviews at three time points were transcribed by the primary researcher and graduate assistants of the project. Each interview transcript underwent a multi-audit/review for accuracy by the research team. A deidentification process was applied, and pseudonyms were created to maintain the participants’ anonymity. 

Three waves of interview transcripts from the four case study participants were reviewed, and text segments that represented general a priori codes related to Asian American university students’ experiences during the pandemic were highlighted. A priori codes were established based on the current study’s qualitative research question. Highlighted text segments were analyzed, and code labels that described and captured the meaning of the highlighted text segments were assigned. In total, 20 code labels were initially extracted, which were combined and collapsed into 12 themes across four common categories. 

The current study’s qualitative method explored the participants’ unique lived experiences, and co-construction was utilized to interpret the findings in order to gain a clear sense of the data [53]. The researcher bracketed her own experiences in order to engage in a fresh perspective towards the phenomenon of interest of the current study [51]. Due to her own experiences as an Asian American university student during the COVID-19 pandemic, the researcher adopted a curious standpoint, understanding that lived experiences are unique and different for each person [54]. Especially for wave three data collection and throughout the data analysis, the researcher reflected on her own experiences, observations, and reflections [52].

#### 3.2.1. Experiences and Events during the Pandemic

This category and its four themes explored the participants’ personal experiences and processing of events that occurred during the COVID-19 pandemic. As Charlotte described, there were a lot of events that occurred during the pandemic that impacted the participants’ day-to-day experiences and state of mind, including the pandemic itself, Trump’s rhetoric, and the 2020 presidential election, as well as racial tension, discriminatory events, and incidents of anti-Asian violence. For the participants, these experiences and events highlighted their Asian identity.

Theme 1: Induced Fear for Safety. During the COVID-19 pandemic, the participants experienced a fear for not only health but also physical safety as rates of anti-Asian hate crimes increased across the country. During the early stage of the COVID-19 pandemic, when wearing a mask was a relatively new practice in the United States and not yet widely mandated or accepted, wearing a mask, although meant to protect oneself and others from the virus, was a major source of worry for the participants as people with Asian complexion. As the reporting of physical attacks on Asian people increased around the country, Amy and Mari indicated an increased effort to protect themselves from being attacked. The participants reported the levels of fear they experienced being in part dependent on their immediate surroundings, such as their and their family’s locations. Mari stated, “I’m very scared to go out in public as an Asian American woman wearing a mask”. Amy also stated, “I think especially because I am Asian and there is so much xenophobia and attention going on that I sometimes am scared to go out. Even though my state has mandated that all people who go to stores have to wear masks, sometimes I’m afraid of how White people will treat me, or if they’ll say something to me or do something to me when I’m out in public…”.

The changes in mask mandates also imposed different meanings on the participants. Amy shared that wearing a mask after the mask mandates were lifted became less so for protection against the virus, and more so for protection from getting discriminated against or attacked because of the way she looks as an Asian person. Towards the beginning of the pandemic, Angel mentioned, “It’s definitely been stressful with the association of all Asians with the virus”. Later, describing his overall experiences during the pandemic, Angel discussed how he was not surprised anymore to experience discrimination due to the increasing number of incidents and the long-lasting and ongoing race-related issues that had occurred over his lifetime. 

Theme 2: Experienced Discriminatory Encounters. The participants not only shared their feelings of unease and fear due to xenophobic attitudes and incidents occurring across the country during the pandemic, they also discussed their personal encounters with anti-Asian discrimination. Angel discussed having felt targeted and profiled prior to the pandemic and then experiencing COVID-19-fueld discriminatory incidents. He said, “I’ve personally had two [encounters of COVID-19-related discrimination]. One of them [in March 2020], when I was in public waiting in line, this random person suddenly saw me and started shouting and pointing at me, saying I had the virus…”.

Amy and Mari shared similar experiences in which they felt targeted based on their race and racial appearance. Their encounters caused them to compare and think about their own experiences with the ones being reported in the news. As Amy stated, “After those two experiences, I’m not necessarily more afraid, but I’m honestly just tired of people making judgments about me just because I look Asian. Those two experiences reminded me that we as a society and as a country have a long way to go”.

Theme 3: Impact of the Black Lives Matter Movement (BLM; 2020). After the death of George Floyd at the end of May 2020 as a result of police brutality, Black Lives Matter (BLM) protests and rallies occurred throughout the country, and ignited a fight against police brutality and discourse on racialized experiences and the experiences of Black, indigenous, and people of color (BIPOC). Angel mentioned that during this time, the lack of initiative and understanding of issues surrounding diversity, equity, and inclusion at his school were highlighted, although racism had always been a “really hot topic”. According to Angel, it was a common belief among the student body that professors did not believe that racism and inequity were a problem there. 

When discussing the overlap between the BLM movement and the anti-Asian sentiment during the pandemic, Angel said, “I’m not sure to what extent [BLM and speaking against Asian xenophobia] have affected each other, but it’s definitely intertwined. I would say generally, the circle of people who support BLM are overlapping with the people who think the profiling and discrimination against Asians due to COVID is a problem. They’re pretty similar in that both groups probably think that discrimination based on race or racial disparities and harassment are wrong”. Similarly, Mari mentioned that people of color all face degrees of discrimination, harassment, and racism. Charlotte and Amy indicated that the reignition of the BLM movement during the pandemic seemed to have steered the public’s attention away from anti-Asian targeting.

Theme 4: Aftermath of the Atlanta Spa Shooting Incident. In March 2021, a shooting spree occurred in Atlanta, Georgia, across three spas/massage parlors. Eight people were killed, six of whom were Asian women. This incident occurred in the midst of other attacks on Asian people across the country and was highlighted by the participants. In discussing the incident, Mari spoke about what she learned about herself and the diversity within the Asian community in processing the Atlanta spa shooting incident (e.g., middle-class student versus undocumented worker). Mari said, “It was certainly traumatic… And I learned a lot from [the Atlanta incident] about the intersection of race, gender, class, and immigration status. I am an Asian woman, but it also made me realize there is so much to that. My life as a middle-class grad student is very different from the life of an undocumented massage worker. It also made me think about the diversity in the Asian community. It’s not just middle-upper class Asian Americans. There are also undocumented workers on the margins…”.

The participants also shared a sense of shock when discovering that people of other races did not quite feel the severity of the anti-Asian hate reflected through these events and the associated fear that they felt. Charlotte also discussed a feeling of frustration with regard to the recurrence of shooting and unchanged legislation. She said, “I do feel very angry and very frustrated. The annoying thing is, in part, there is no way to express and let out the anger and the frustration in a productive manner. The anger… just so many…”.

#### 3.2.2. Categorization of Asians in America

This category and its two themes explored how participants experienced others’ perception of Asian Americans, Asian people, and people of color in general in America, as well as the effects of being considered under one broad category. Although these experiences did not all occur solely during the pandemic, they were experiences that informed the participants’ processing and understanding of their experiences during the pandemic.

Theme 5: Othering of Asian Americans. The participants referred to the experience of being “othered”. Othering is an imposed phenomenological concept that describes how a group of people, minority, or less powerful groups, such as Asians in America in this context, are perceived as outsiders and often inferior. For example, othering can be experienced via microaggression, which can be a statement, action, or incident regarded as an instance of indirect, subtle, or unintentional discrimination against members of a marginalized group. Despite Angel’s true background and his identification as an American, he is cognizant that people do not perceive him as that solely because of his Asian complexion. He further explained that it is a common experience for Asian Americans to be asked where they are from and that the people asking are often not satisfied without an answer of somewhere in Asia. He said this situation is “definitely not something that, for example, White people [experience]. If someone said they’re from Wisconsin, people wouldn’t be like, ‘But where in Europe are you from’ or ‘Where are your parents from’ because they’re accepted as the dominant majority [here]”. 

Theme 6: Umbrellaing—People of Color. Umbrellaing is to group a wide range of concepts, or in this case, people of different races or ethnicities, under one category. However, umbrellaing does not accurately depict the relationships of the different subgroups within a broad category. The participants discussed the experience of this phenomenon both in terms of a broad “people of color” category and a broad “Asian” category. Mari expressed that although umbrellaing can unify people from diverse backgrounds who have similar experiences and plights, this is not always appropriate. For example, Mari and Angel mentioned that “Chinese” is often taken to be synonymous with “Asian”, even though there are so many more different Asian countries and identities. Charlotte highlighted the separation that exists among Asian people, stating, “It’s from the Caucasian point of view that Asian Americans get along. If you ask a Chinese American, Chinese American is Chinese American and Korean American is Korean American”.

#### 3.2.3. Categorization of Asian Discrimination

This third category and its three themes explored ways to improve the stereotypical perceptions of and discriminatory outlook against Asians and Asian Americans. Due to their experiences throughout the pandemic and as Asian Americans in general, the participants had thoughts and desires for ways to fight anti-Asian sentiment. There are also general stereotypes and expectations for Asians that have impacted the participants’ experiences, and they discussed how they processed these in light of the events and their experiences during the pandemic.

Theme 7: Exploring Asian Stereotypes and Expectations. Common stereotypes for Asian Americans, particularly Asian women, but also Asians in general, include being demure, shy and quiet, obedient, and not outspoken. Amy highlighted that these characteristics are actually embodied by the Asian population, but that she believed that the Asian population should speak out “because in the end, it’s the minority population overall that do not have the same power in their vote”. Mari mentioned that Asian stereotypes have impacted her behavior in the classrooms, while Charlotte stated, “We have always been kind of hidden in the background where we do good work and have the white-collar jobs, or [we are seen] as a good resource to have around”. Although, through the pandemic experiences, Amy began to not pay as much attention to what others think, meeting the stereotypical expectations has been a source of inner conflict for her in general, as she does not feel that she receives recognition for her hard work since that is what is expected from her. 

Another factor regarding perceptions of Asians, the fetishization and sexualization of Asian women, was particularly exposed after the Atlanta spa shooting incident, which Mari said made her reflect more deeply on her personal experiences from a time when she Googled herself in high school and came across a website that was “clearly a site to sexualize and stalk Asian women” and that included their Facebook profiles, the events they attended, and their locations. Mari said, “At the time I thought that’s really weird and disturbing, but I didn’t think of it after that. Now looking back I’m like, wow, this is definitely an Asian woman thing, being sexualized and taken as very interchangeable with other Asian women”. 

Theme 8: Combatting Discrimination Against Asians. As Angel said, “Even before the pandemic, there was already a lot of racism and discrimination and invisibility of Asian Americans”. However, the anti-Asian rhetoric and sentiment during the early stage of the COVID-19 pandemic and subsequent racist statements, violent actions, and hate crimes brought up a different experience with Asian discrimination for the participants. Charlotte discussed the importance of unifying and speaking up against social prejudice early on. With regard to combating anti-Asian sentiment and teaching people to treat each other equally, Amy highlighted that it should be “a wide-spread mass movement and universal change of thinking”. 

Although some participants were optimistic about the changes occurring, Angel feared that the anti-Asian sentiment associated with the pandemic might have lasting impacts. Mari, on the other hand, highlighted that it is important “to distinguish between the government of a country and the people and diaspora that are associated with it”.

Theme 9: The importance of Representation. Good, accurate, and widespread representation of Asian Americans is a direct way to address anti-Asian sentiment, as well as the general population’s reliance on stereotypes, and to increase familiarity with the Asian population. Angel spoke about the importance of there being better representation so that it could increase the chances of Asian populations being heard, provide opportunities for people to interact with others from different backgrounds, and enable more open-minded people to hold “less preconceived notions about others”. Specifically, Charlotte highlighted the importance of portrayal of each race in the media, as such portrayals could contribute to the assumptions and associations that the general public may have towards another race. Representation and presence in the media is important as it can affect people’s sense of belonging. Charlotte said, “It never really hit me how important a community is until the last two years, how important it is to have representation, how important it is that I can go to work and see somebody that looks the same as me or watch TV and see characters I can relate to”. Charlotte shared that her experiences during the pandemic made her realize that due to the lack of representation, she does not feel that America is her home and that she relates to others here.

#### 3.2.4. Renewed Sense of Asian Identity

This final category and its three themes explored how participants’ attitudes and approaches towards themselves and others changed, considering their overall experiences, especially those throughout the pandemic. Each of the participants found that the way they approached the world and their interactions with others changed. Their sense of self, their identity, and the way they perceived others and their experiences were impacted. 

Theme 10: Finding Community and Connection. All participants discussed that the pandemic period shed light on the importance of community. To process current events, their own experiences, and their fears, and to understand how to move forward, the participants found support in their friends and peers, particularly those who were also Asian or Asian American. Angel stated, “I had to seek support from my friends and tell them about it because I realized it was better for me to share my experience rather than keep them to myself and feel worse about myself. It helped when people affirmed and supported my feelings”. The pandemic and the associated anti-Asian sentiment brought the participants closer to their social networks. The participants also relied on their peers outside of their families to process tension within their families regarding the topic of race. Although the participants felt more empathetic and understanding of the history and experience of Black people in America because of the BLM movement, which occurred during the pandemic, their family members did not have the same perspectives. 

Theme 11: Behavioral Changes. Through their experiences during the pandemic, the participants found that the focus of their time and energy shifted. Angel stated, “I started reading more about Asian American figures, celebrities, and history. [The pandemic experiences] made me seek out more Asian American people and experiences, even if it just was through social media or the media, just to hear people’s perspectives”. The participants found meaning in understanding others’ experiences, in finding belongingness in a community, and in using their own voice. Connecting with others who were like them and had similar experiences, with dialogue and conversation, helped participants find their way through the pandemic. The participants also discovered the importance of actively hearing the experiences of others, as well as using their own opinions and voice more politically to make changes for the better.

Theme 12: Redefined Identity. The participants shared that being able to find positivity in their experiences during the pandemic was a way of coping. Each participant described how their sense of self changed during the pandemic period. Charlotte described how she had the time to reflect on what she is and who she is. Angel reflected on his stronger sense of identity as an Asian American as a way to connect and empathize with others across minority groups. Mari spoke about how her experiences during the pandemic reaffirmed her identity as an Asian American and helped her reconnect with culture and traditions. Amy shared that she had recently discovered that she did not need to be so consumed with others’ perception of her. Conversely, Charlotte’s sense and understanding of her identity were intertwined with her sense of belonging, which did depend on how she was perceived and accepted by others. Based on her background of being an Asian American who spent part of her childhood growing up in China, Charlotte felt as though she does not quite belong to any group of people.

## 4. Discussion

This study shed light on the experiences of Asian American university students during the COVID-19 pandemic and explored how those experiences affected their view of themselves as Asian Americans. The four case studies presented provided insight into four categories of themes. From the interviews with the participants, 12 key themes came to light that aligned with the extant literature on Asian American identity and experiences. 

Although the Asian American Racial Identity Development model (AARID) [46,47] was intended to describe Asian American’s identity development throughout their lives, the case study participants’ accounts of their experiences and reflections during the COVID-19 pandemic depicted the five stages of the model. Each stage of the model includes a shift in self-concept that incorporates evaluation and meaning attribution. Most participants described gaining a positive racial identity through navigation of negative messages and stereotypes about Asian Americans. All participants described feeling a sense of being different during the pandemic. The COVID-19 pandemic was politicized and racialized with charged rhetoric (e.g., “Chinese flu”, “Wuhan virus”, “Kung flu”), which contributed to the public’s xenophobic attitudes and behaviors towards the Asian American community. 

The participants also described, to some extent, experiencing an “awakening to social political consciousness” through increased political awareness and a minority-oriented association and perspective. They mentioned a “redirection to an Asian American consciousness” through their social experiences and connections. As part of this stage of the model, Asian Americans experienced anger and outrage toward the country’s dominant majority for the acts of racism directed towards Asians. In processing their experiences during the pandemic, the participants reframed their understanding of their overall experiences as Asian Americans, reaffirming previous research that Asian Americans have a unique minority experience, as they are both labeled the “model minority” and are seen as perpetual foreigners, continually perceived as outsiders and a threat, which was exacerbated during the COVID-19 pandemic [55,56]. Furthermore, while Asian Americans are perceived as homogenous, it is important to distinguish among individual subgroups within a category. Grady [57] described in an article highlighting commentary from sociocultural linguistics that the umbrella term “people of color” groups all non-White people together in a way that is not necessarily productive, as it does not give each subgroup its own attention. Umbrella terms such as “people of color” and “BIPOC” suggest unity, solidarity, and shared positionality among all people of color. Although aspects of the experiences of different groups of people of color may be similar, as the current study’s participants discussed, especially in their processing of the 2020 BLM movement, the suggested unity is not always accurate and it disregards the differences and uniqueness of each subgroup [57,58]. This false sense of unity is also the case for the umbrella term “Asian American”.

According to the AARID model, Asian Americans go from a reactionary state to feeling secure and, with support, reflecting on their own experiences and immersing themselves in Asian American history and culture. The participants in this study discussed the ways in which they processed the events of the pandemic with their social support circles and how finding other Asian Americans who had similar experiences was important in their coping. The participants also considered ways to confront the overall stereotyping of Asian Americans and anti-Asian sentiments, and narrated their reflection of figuring out “what parts of themselves are Asian and what parts are American” [46,47]. Three of the four participants confidently reached the “incorporation” stage of the AARID model during wave three of the interviews, expressing a sense of confidence in their Asian American identity. All participants discussed some shifts in their behavior and sense of self. It is evident that Asian American identity is influenced by social contexts and social interactions [46,47,48,49]. 

The media can impact the experiences of Asian Americans, influencing their adjustment, socialization, and identity development [59,60]. PBS NewsHour Student Reporting Labs emphasized that a lack of minority representation in the media can have an impact on the self-esteem, mental health, and identity formation of individuals within the population [60]. The current study similarly highlighted the importance of media representation and representation in general of the Asian American population to foster a sense of belonging and combat stereotypes. Future research can consider specifically exploring the impacts on Asian Americans’ sense of belonging and ability to relate to cultural contexts, as well as on their overall identity development as Asian Americans. 

Although the current study did not compare Asian American participants to individuals from other racial and ethnic groups, differences in perceived discrimination or mistrust among different racial/ethnicity groups are reported in other empirical studies. For example, Morgan et al. [61] reported that, in comparison to White non-Hispanic individuals, Black non-Hispanic/Black Hispanic/White Hispanic individuals had lower odds of having the COVID-19 vaccine, after accounting for medical mistrust. The relationship between race and ethnicity and having the COVID-19 vaccine was mediated by the combined perceived discrimination and medical mistrust. Sherchan et al. [62] further explained the perceived COVID-19 severity and susceptibility among Latent Class 1 members. Sherchan et al. [62] reported that students in Latent Class 1 reported higher perceived COVID-19 threat (perceived COVID-19 susceptibility) compared to students in all other latent classes, and the students in Latent Class 1 were more likely to identify as Hispanic or Latino, non-Hispanic Asian, non-Hispanic Black or African American, and non-Hispanic Multiracial compared to non-Hispanic White. In short, the findings of the current study concur with those of other researchers in terms of the importance of examining perceived susceptibility and discrimination among minority individuals. As explained earlier in this paper, the current study recruited four participants from a larger sample. The original larger-scale study quantitatively examined Asian American university students’ adjustment, coping, and stress during the COVID-19 pandemic [63]. The Asian American student sample reported lower academic adjustment compared to the non-Asian American student sample during the COVID-19 pandemic. The Asian American sample reported significantly lower use of escape avoidance coping than the non-Asian American sample [63]. Among all of the 103 Asian American students who were recruited for the original study, four remained available for wave one, wave two, and wave three interviews (the current study). Coupled with the qualitative findings of the current study, it is important to examine Asian Americans’ unique experiences and reactions to the COVID-19 pandemic. 

## 5. Limitations 

This study specifically examined the experiences and identity of Asian American students in relation to the COVID-19 pandemic. As highlighted by the case study participants, those who identify as Asian American are a very diverse population. There are as many as 25 different Asian American ethnic groups [64,65]. However, generalizing all Asians under one category is particularly problematic, considering their varying diverse backgrounds, different histories, cultures, values, languages, and so on. Each subgroup of Asian Americans comes with unique histories, cultures, languages, and other characteristics. Due to the network of the researchers, who are mostly Asian Americans or Asians originally from the Far East of Asia, the participants that were recruited were those with Chinese or Taiwanese backgrounds, which largely limited the generalizability of this study. The U.S. Census Bureau also excluded Asian American Pacific Islander communities from “Asian” despite the number of hate crime reports targeted at these communities. Future research can examine differences in discriminatory impact and adjustment, as well as overall experiences during the COVID-19 pandemic, of those who identify as other East Asian (e.g., Korean, Japanese) and Southeast Asian populations, Asian American Pacific Islander communities, and international Asian students in the United States.

The original larger-scale study recruited 517 participants through convenience sampling, allowing for sampling bias [66]. The four case study participants were from the original study, and they voluntarily participated in three phases of interviews, highlighting the potential that they may have had different motivations or accessibility than those who did not express interest in interviews (e.g., time and availability to sit for a lengthy interview, technology accessibility). Moreover, the participants themselves chose to potentially participate in follow-up interviews; therefore, the participants who selected themselves may have been more willing to be expressive and communicative about their experiences during the COVID-19 pandemic. Interviews were conducted virtually in accordance with IRB approval, using only audio communication. Conducting interviews in this manner allowed for the participation of those in different states and across the country and provided a sense of security for the participants. However, the data collected lacked insight and information that might have been revealed through nonverbal communication cues that would have been observed in-person or over video. Future researchers might consider in-person interviews to better capture the non-verbal communication cues and body language of the participants. Moreover, limitations related to research protocol timelines, as well as the usage of secondary data, may have affected the timeliness and richness of data. The case study participants’ in-the-moment experiences and reflections may not have been fully captured at the time of the interviews. For example, wave one and two interviews of the original larger-scale study were not conducted to specifically explore how Asian American university students’ experiences during the pandemic might have impacted their identity. Wave three interviews were conducted months after the Atlanta spa shooting incident and other prominently reported attacks on individuals in the Asian community. Future research should seek to address the limitations of this study’s methodology in an attempt to capture participants’ experiences, reactions, and behaviors more accurately or completely.

## 6. Conclusions and Implications

This study explored the unique experiences of Asian American undergraduate and graduate students during the COVID-19 pandemic and the impact of their experiences on their identity. The results suggested that the COVID-19 pandemic-associated anti-Asian sentiment and racist acts of hate and incidents of violence had a great effect on Asian American university students’ experiences and overall processing of events during the COVID-19 pandemic. Future research should continue to explore the unique experiences of Asian American university students and their identity and growth in order to contribute to higher education research on this population, as well as the Asian American population in general. The current study contributes to the psychological literature as it can inform potential and necessary support and interventions to improve students’ experiences during unprecedented situations, such as the COVID-19 pandemic.

The Asian American experience of the COVID-19 pandemic was unique compared to that of non-Asian Americans. This study suggested that the COVID-19 pandemic-associated anti-Asian sentiment, xenophobia, and racism impacted Asian Americans’ overall experiences, coping, stress, and adjustment during the pandemic, in addition to the related health concerns. 

Asian American identity is influenced by social contexts, relationships, and external forces [47,49]. This study’s participants described their experiences and the events of the pandemic, as well as their overall experiences as Asian Americans. Overall, through their navigation and processing of their pandemic experiences, they found a new understanding of their identity as Asian Americans. By using active coping, through processing of and reflecting on experiences, as well as changing their approach towards others through their attitude and behavior, the participants were able to find hope for themselves to move forward. Mental health professionals can consider the benefits of positive reappraisal coping when clients face an intense and widespread stressor, such as the COVID-19 pandemic and its associated anti-Asian rhetoric and discrimination toward Asian Americans. 

The vulnerability of Asian Americans during the COVID-19 pandemic was compounded by the pandemic-related anti-Asian sentiment. Even those who were not personally subjected to acts of hate and violence experienced mental fatigue and exhaustion, in addition to the overall stress and anxiety caused by the COVID-19 pandemic [14]. Pandemics can further marginalize already marginalized groups by intensifying the social boundaries between identity groups via rhetoric placing blame and the naming of pandemics (e.g., “China virus”, “Kung Flu”), such as intensifying the “othering” felt by the participants of the current study. Dhanani and Franz [4] explored the language associated with describing the virus and the pandemic and found that emphasis on the connection between China and COVID-19 increased negative attitudes towards Asian Americans, whereas emphasis on the serious health risks of COVID-19 was not correlated with increases in bias against Asian Americans or xenophobia. Public health professionals, journalists, and government officials, as well as the general public, should be mindful of the language used when discussing disease outbreaks and epidemics. 

The current study examined college students and graduate students; thus, it is suggested that some intervention strategies can be applied in higher education. For example, at the university level, university counseling centers can provide online workshops and focus groups to address perceived challenges and discrimination among students. At the school/program level, accommodation can be provided to a certain student population who might be particularly vulnerable during the pandemic. At the individual level, academic advisors should be mindful of perceived challenges and discrimination among students, especially those from culturally and linguistically diverse backgrounds. 

For the case study’s participants, finding a community and a network of people with similar experiences to process the events during the pandemic was important for navigating their fear and stress, as well as for overall coping and adjustment. The availability of campus social networks and spaces helps foster a sense of community among students, as well as the development of critical consciousness and transformative resistance in students from marginalized groups [48]. University institutions should support spaces for Asian American students to engage in such dialogue, explore conflicts and connections, and develop an understanding of how to navigate their experiences. Currently, such spaces are few and far between, reaching few Asian American students, or are limited in that they focus on marginalized groups generally (i.e., BIPOC) and do not allow for group-specific processing. 

Additionally, during the COVID-19 pandemic, Asians were not the only identified targeted group [10]. Academic staff and mental health professionals should be conscious of their own responses to their students’ and clients’ experiences, especially those of marginalized groups, and find effective ways to connect and support them as they navigate their experiences. The longitudinal findings of the current study indicated that the participants’ perceived challenges and discrimination changed and evolved over time. Thus, university-based mental health providers and academic advisors need to be mindful of the changing circumstances associated with the public health emergency, such as COVID-19 in this study. 

## Figures and Tables

**Table 1:** Qualitative participant demographic and background information (N = 4).

“Pseudonym” (Age), Sex, Asian Ethnicity	Salient Personal Information
“Charlotte” Female, Chinese, second-generation, private university (graduate program)	Charlotte is a graduate STEM student pursuing a degree in mechanical engineering. Charlotte resides outside the New York City metropolitan area, within a suburb in the Greater Boston metropolitan area. During the spring 2020 semester at the start of the pandemic, she was in her second year of her program, finishing her master’s degree. She began pursuing her PhD in the fall of 2020 at the same university. She is a second-generation Asian American who is bilingual in English and Chinese. She grew up in China for a period of her childhood for about 7 years, from age 3 years to about 10 years. She has a family abroad living in China. At the onset of the pandemic, Charlotte was living off-campus. Throughout the pandemic, she remained in the state of her university and lived with her extended family and roommates. Her parents are divorced, and she, her parents, and her sister live in different states across the country. During the 2020–2021 academic year, she attended classes remotely, while her university implemented a hybrid modality, and she attended lab in-person. For Charlotte, some important events during the pandemic included finishing her master’s program, having graduation pushed from May to August as she finished her thesis, and starting a PhD program. Access to resources, equipment, and materials, as well as collaboration with peers and the availability of professors, were limited during the pandemic. Additionally, the reporting of pandemic-related news and of discriminatory events was overwhelming for Charlotte, and the content was frustrating for her to digest. Other salient points for Charlotte during the pandemic included the inauguration of President Biden and the January 6 Capitol Riot.
“Mari” Female, Chinese, second-generation, private university (graduate program)	Mari is a graduate student pursuing a doctoral degree in psychology on the West Coast. At the start of the COVID-19 pandemic, she was in her second year of the program, which is located outside of the New York City metropolitan area and within the Bay Area of California. Her school continued to implement a remote modality in the 2020–2021 academic year. Mari is a second-generation Asian American who is bilingual in English and Chinese. She has a family living abroad in China. Her parents are divorced and live on opposite coasts of the United States. She also has a younger paternal brother who is six years old. Growing up, Mari was very close to her mother. Throughout the pandemic, she had to reconsider her family responsibilities towards her mother as Mari learned to navigate how to communicate with and support her mother emotionally, as they had differing views and as Mari continued to grow on her own as an independent individual. For Mari, some important points during the pandemic included working through the uncertainty of all aspects of her life due to the pandemic. Prior to the start of the pandemic, Mari’s long-distance partner, who was located in another state, was planning to move in with her into campus couples’ housing and had already ended his lease and left his job. However, their plans were disrupted when the pandemic hit and the university campus closed, which also impacted her research work. Her mental health was taking a toll, so she decided to join her partner where he was instead. They were able to extend the lease and settle together in the Chicago metropolitan area. After a period of time, they moved together back to where her university is located and moved in with some of her friends. Other salient points for Mari during the pandemic were the election season and the Atlanta spa shooting incident.
“Angel” Male, Taiwanese, second-generation, private university (nursing program)	Angel is a graduate student pursuing a master’s degree in nursing at a university located within the New York City metropolitan area in Connecticut. At the start of the pandemic, he was in his first year of the program. He is a second-generation Asian American who is multilingual and has varied language abilities in English, Mandarin, Spanish, and French. He has a family abroad living in Taiwan. At the onset of the pandemic, Angel was living off-campus with roommates who were away for spring break and who had decided to stay home and be with their families instead of returning to their apartment during the initial pandemic period. Angel found being socially isolated difficult, as it was impacting his mental health. During the 2020–2021 academic year, Angel’s university implemented a hybrid modality, although his schedule was heavily remote. He preferred in-person learning and experiences, but he could foresee increasingly enjoying hybrid schedules in the future. He found benefits to attending classes virtually as the pandemic continued after he moved into another apartment and had more space: a hybrid modality would allow for in-person experiences as well as flexible scheduling that can include being able to log off and spend time outside. For Angel, health and racial issues were important areas of concern during the pandemic. He was frustrated by the unwillingness of people in the United States to adhere to health and safety precautions, such as the spread of misinformation and the politicization of wearing masks. Angel also highlighted the issue of racial and health disparities. He also stressed the scapegoating and othering of minority groups, particularly Asians, in America. Additionally, during the pandemic, there was increased attention on Asians and Asian Americans, resulting in hate crimes and racial prejudice. Being bombarded with news about the pandemic and events of discrimination against Asians was stress-inducing for Angel.
“Amy” Female, Chinese, second-generation, private university (undergraduate program)	Amy is an undergraduate STEM student studying computer science. Amy resides in the New York City metropolitan area. At the start of the pandemic, she was in her first year of the program. She is a second-generation Asian American who has native proficiency in English and conversational proficiency in Mandarin. She has a family living abroad in China. She grew up in a suburban neighborhood that did not have much of an Asian population presence. At the onset of the pandemic, Amy was forced to move out of her university dorm and back home with her family in a state different from where her university is located, and she lived with her parents and younger sibling, who is in middle school. As the 2020–2021 academic year started, she moved back to the city and state where her university is located, while she continued to attend classes fully remotely. For Amy, a defining factor of the pandemic time was navigating the social distancing routine. Remote learning was difficult for her, and her mental health took a toll due to her monotonous schedule during the pandemic, not being able to have her typical lifestyle of moving from place to place throughout her days, as well as the lack of person-to-person interactions. Other salient points for Amy during the pandemic included the inauguration of President Biden, the January 6 Capitol Riot, the Chauvin trial, and moving back to the city and living on her own.

## Data Availability

Due to privacy concerns mentioned in the IRB protocol, the data associated with this study cannot be provided to the public without the supervision of the researchers. However, individual researchers who are interested in obtaining access to the data for individual use are encouraged to contact the corresponding author.

## Appendix A. Interview Guides for Wave One, Wave Two, and Wave Three

Wave One

*Follow-up as needed based on participants’ responses, keeping in mind pre-existing conditions, school, family, living situations, areas of support, thoughts/experiences related to racism, etc*.

1. Could you share the major difficulties that you encountered during the COVID-19 pandemic?
2. Could you share the major challenges that you encountered during the COVID-19 pandemic?
3. Have you encountered any COVID-19-related discrimination, hate crime, or unpleasant experiences, etc.? If so, how do you feel and how do you cope?
4. What would you like to see in terms of support at the community level?
5. What would you like to see in terms of support at your university level?
6. What would you like to see in terms of support at your school level?
7. What would you like to see in terms of support at your program level?
8. Is there anything else you would like to share?
9. Are you interested in a 6-month follow-up interview?

Wave Two

*Follow-up as needed based on participants’ responses, keeping in mind pre-existing conditions, school, family, living situations, areas of support, thoughts/experiences related to racism, etc.*

1. Could you share your major difficulties and challenges you have encountered or are currently facing because of the COVID-19 pandemic since the last interview?
2. Could you share if you have experienced any positive changes during or because of the COVID-19 pandemic?
3. Could you share your thoughts about the safety protocols and regulations that have been enforced (e.g., quarantining after travel, physical distancing, wearing masks)?
4. During the last interview, we asked if you experienced any COVID-19-related discrimination, hate crime, or unpleasant experiences. COVID-19 fueled anti-Asian racism and xenophobia worldwide. Have you had any such experiences since the last interview? Were you aware of this happening?

*Follow-up with question(s) about how the Black Lives Matter movement affected this issue of Asian xenophobia: Due to current events that happened during the past few months, discrimination based on race has been a hot topic in our country especially. How do you think the Black Lives Matter movement affected this issue of Asian xenophobia?*

5. (A) For students: What is your school’s current operation (in person, 100% remote, hybrid)? What did you think of the plan when it was announced? When was it announced? How is it going? How do you feel about any regulations that have been enforced? What types of support are provided? What would you like to see in terms of support from your university and program? Is there any discussion about steps forward/the Spring semester?
  (B) For non-students who graduated during Spring 2020: Was there/is there support you received related to job hunting during the pandemic? What is your job’s current operation (in person, 100% remote, hybrid)? How is it going? How do you feel about any regulations that have been enforced? What types of support are provided? What would you like to see in terms of support? Is there any discussion about the steps forward?
6. Is there anything else you would like to share?

Wave Three

1. When you reflect on this past year of the COVID-19 pandemic, what are some salient points and events that were important to you?
2. How have your relationships with others changed due to your experiences during the pandemic year?
3. What are your thoughts when reflecting on the COVID-19 pandemic reportedly starting in China, which led leaders to use phrases such as Wuhan or the China virus, implying blame for the pandemic? How did you navigate this?
4. On 16 March 2021, 8 people were killed in the Atlanta-area massage parlor shootings, and 6 of the victims were Asian. The Atlanta shooting incident occurred in the midst of other attacks on Asian people across the country. Asian people were being randomly targeted, and footage of these attacks was shared on the news and through social media. How did you experience this time period?
5. Do you have any questions for me, or is there anything else you would like to add?
